# The iSplit GFP assay detects intracellular recombinant proteins in *Bacillus subtilis*

**DOI:** 10.1186/s12934-021-01663-7

**Published:** 2021-09-06

**Authors:** Patrick Lenz, Fabienne Hilgers, Alina Burmeister, Leonie Zimmermann, Kristina Volkenborn, Alexander Grünberger, Dietrich Kohlheyer, Thomas Drepper, Karl-Erich Jaeger, Andreas Knapp

**Affiliations:** 1 Institute of Molecular Enzyme Technology, Heinrich Heine University Düsseldorf, Forschungszentrum Jülich, 52425 Jülich, Germany; 2 Institute of Bio- and Geoscience, IBG-1: Biotechnology: Forschungszentrum Jülich GmbH, 52425 Jülich, Germany; 3 RWTH Aachen University, Microscale Bioengineering (AVT.MSB), 52074 Aachen, Germany; 4 Multiscale Bioengineering, Bielefeld University, 33615 Bielefeld, Germany; 5 Present Address: Castrol Germany GmbH, 41179 Mönchengladbach, Germany

**Keywords:** *Bacillus subtilis*, iSplit GFP assay, Intracellular protein, Online monitoring, β-glucuronidase, Flow cytometry, Microfluidics

## Abstract

**Background:**

*Bacillus subtilis* is one of the most important microorganisms for recombinant protein production. It possesses the GRAS (generally recognized as safe) status and a potent protein secretion capacity. Secretory protein production greatly facilitates downstream processing and thus significantly reduces costs. However, not all heterologous proteins are secreted and intracellular production poses difficulties for quantification. To tackle this problem, we have established a so-called intracellular split GFP (iSplit GFP) assay in *B. subtilis* as a tool for the in vivo protein detection during expression in batch cultures and at a single-cell level. For the iSplit GFP assay, the eleventh β-sheet of *sf*GFP is fused to a target protein and can complement a detector protein consisting of the respective truncated *sf*GFP (GFP1-10) to form fluorescent holo-GFP.

**Results:**

As proof of concept, the GFP11-tag was fused C-terminally to the *E. coli* β-glucuronidase GUS, resulting in fusion protein GUS11. Variable GUS and GUS11 production levels in *B. subtilis* were achieved by varying the ribosome binding site via spacers of increasing lengths (4–12 nucleotides) for the GUS-encoding gene. Differences in intracellular enzyme accumulation were determined by measuring the GUS11 enzymatic activity and subsequently by adding the detector protein to respective cell extracts. Moreover, the detector protein was co-produced with the GUS11 using a two-plasmid system, which enabled the in vivo detection and online monitoring of glucuronidase production. Using this system in combination with flow cytometry and microfluidics, we were able to monitor protein production at a single-cell level thus yielding information about intracellular protein distribution and culture heterogeneity.

**Conclusion:**

Our results demonstrate that the iSplit GFP assay is suitable for the detection, quantification and online monitoring of recombinant protein production in *B.* *subtilis* during cultivation as well as for analyzing production heterogeneity and intracellular localization at a single-cell level.

**Graphic abstract:**

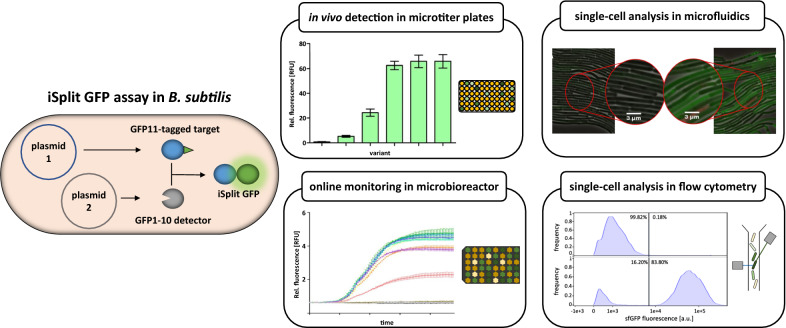

**Supplementary Information:**

The online version contains supplementary material available at 10.1186/s12934-021-01663-7.

## Background

The Gram-positive soil bacterium *B. subtilis* is one of the most important microorganisms for industrial protein production [1]. It has some major advantages over the commonly used Gram-negative *Escherichia coli.* As a Gram-positive bacterium, it does not produce endotoxins such as lipopolysaccharides, which is one of the reasons that *B. subtilis* has earned the GRAS (generally recognized as safe) status by the US Food and Drug Administration (FDA) [2]. In addition, *B. subtilis* has an unbiased codon usage, allows fermentation in large quantities, and genetic manipulation is easily possible by natural competence [1, 3, 4]. It is well known especially for the secretion of recombinant target proteins or enzymes due to its large secretion potential allowing for yields of up to 20 g/L [1]. Consequently, numerous efforts have addressed the optimization of protein production and subsequent secretion [5–10]. A large set of expression systems and expression host strains are available (for reviews see ref [11, 12]). which enable efficient production of recombinant proteins in *B.* *subtilis*.

The pertussis toxin subunit S4, for example, was probably one of the first pharmaceutically important proteins produced intracellularly in *B. subtilis* [13]*.* Today, industrially relevant enzymes are still produced in the cytoplasm of *B.* *subtilis*, for instance the trehalose synthase TreS from *Pseudomonas putida* ATCC 47054, which catalyzes the reversible interconversion of maltose and trehalose, the latter being an industrial sweetener or stabilizer [14].

The detection and quantification of recombinantly produced proteins is key for biotechnological processes. Often, enzymatic activity assays are used in combination with photometric or fluorometric reagents, as for lipases [15], proteases [16], or β-lactamases [17]. If activity assays cannot be performed, activity-independent detection methods including the enzyme-linked immunosorbent assay (ELISA) and Western-blotting in combination with immunodetection have to be used. Preferably, such detection methods should be amenable to a high-throughput format. Recently, we have established a high-throughput assay for the detection of secreted proteins in *B. subtilis* as a universally applicable method independent of enzymatic activity [18]. This method is based on the split GFP assay that has initially been established for in vitro and in vivo detection of soluble target proteins in *E. coli* [19–21]. To detect proteins that were produced and secreted by *B. subtilis*, the eleventh β-sheet of GFP (GFP11) was fused to a target protein and the truncated, non-fluorescent variant GFP1-10 (detector), which lacks the eleventh β-sheet, was subsequently added. Detector and the GFP11-tag of the secreted protein assembled and thus formed a holo-GFP, whose fluorescence can be detected spectrometrically. Besides the detection of target proteins in bacteria, split GFP was further applied for various in vivo analyses including the detection of protein–protein interactions, mitochondrial localization of dual localized proteins, neuronal cell communication and host–pathogen interactions [22–25].

In the present study, we established a split GFP assay for monitoring intracellular recombinant proteins produced by *B. subtilis*. The β-glucuronidase GUS from *E. coli* [26] served as model protein for which an easy-to-perform colorimetric activity assay exists using *p*-nitrophenyl glucuronide as substrate [27]. GUS and GUS11 production was under control of the strong constitutive promoter P_*HpaII*_ [28] and was gradually tuned by a differential translation initiation as described previously [29]. The detector protein was co-produced under the control of the IPTG inducible promoter P_*grac*_ [30] with GUS in *B. subtilis* allowing for detection of cytoplasmic GUS via reconstituted split GFP fluorescence in vivo. This system named intracellular split GFP (iSplit GFP) can also be used to visualize population heterogeneity and intracellular localization of recombinant proteins at the single-cell level as shown by flow cytometry and microfluidic single-cell cultivation in combination with live-cell fluorescence microscopy.

## Material and methods

### Bacterial strains, media and growth conditions

All experiments were performed with the protease-deficient strain *B. subtilis* DB430 [31]. The bacteria were cultivated at 30 °C in enriched LB medium [1% (w/v) NaCl, 8% (w/v) tryptone, 0.5% (w/v) yeast extract] containing either 50 µg/ml kanamycin for maintenance of plasmid pBSMul1 [32] and GUS or GUS11*-*encoding derivatives and/or 5 µg/ml chloramphenicol for plasmid pHT01 ([33], MoBiTec, Germany) and *sf*GFP- or detector*-*encoding derivatives. *E. coli* strain DH5α [34], used for molecular cloning, or *E. coli* BL21(DE3) [35] used for detector production, were cultivated at 37 °C in LB medium [1% (w/v) NaCl, 1% (w/v) tryptone, 0.5% (w/v) yeast extract] containing 100 µg/ml ampicillin. Transformation was carried out using naturally competent *B. subtilis* cells [36] and chemically competent cells for *E. coli* [37].

### Recombinant DNA techniques

Standard DNA techniques were performed as described in [37]. For the purification of plasmids and PCR products, appropriate kits from Analytic Jena (Jena, Germany) were used. Enzymes were purchased from Thermo Fisher Scientific (St. Leon-Roth, Germany).

### Construction of the pBS-Xnt-GUS11 plasmid series

The GFP11-tag-encoding DNA fragment was fused to the 3′-end of *gus* by a 2-step PCR approach (as described in [21] and [18]) using the plasmid pBS-4nt-GUS [29] as a template. For the first PCR step, the primers pBSMul_for (5′ GGAGCGATTTACATAATAAGGAGGACATATG 3′) introducing a NdeI site at the 5′-end, and GUS-rev-fu1 (5′ TGATCACGAGATGTAGAGCCGCCGCCAGAGCCGCCATCAGAGCCGATAAGTTGTTTGCCTCCCTGCTGCGGTTTTTC 3′) were used to add half of the GFP11-tag at the 3′-end. For the second step, the same forward primer was used with Rev-fu2 (5′ TATATCTAGATTATGTGATGCCAGCAGCGTTAACGTATT 3′). With the second step, the remaining part of the tag and an XbaI site were added. By using this approach with split primers, the Rev-fu2 primer can be used independent of the target gene. The final PCR product was then hydrolyzed with NdeI and XbaI and ligated into the likewise hydrolyzed pBSMul1 vector series with spacer lengths varying from 4 to 12 nucleotides [29].

### Construction of vectors for production of sfGFP detector derivatives

For construction of the *B. subtilis* expression vector pHT01-GFP1-10, the already existing vector pET22b-GFP1-10 [18] was used as a template for SLIC cloning [38] of a DNA fragment encoding GFP1-10 into the vector pHT01 using the primer pair 1f-i-pET22b-s-o(Vec) (5′ GGATAACAATTCCCAATTAAAGGAGGAGATATACATATGAGCAAAGGAGAAGA 3′)/1r-i-pET22b-s-o(Vec) (5′ GTATCCTCTAAGTAATATGAATTCCCTTCCAGCCGGATCTCAGTGGT 3′) for amplification of *GFP1-10* gene and the primer pair Vf-pHT01 (5′ GAAGGGAATTCATATTACTTAGAGGATACT 3′)/Vr-pHT01 (5′ CCTCCTTTAATTGGGAATTGTTATCCG 3′) for amplification of the pHT01 vector. For obtaining pHT01-iSplitGFP, a stop codon was introduced into the pHT01-sfGFP vector [39] by exchanging CGT with TGA at base pair positions 643–645 via QuikChange® PCR [40] with the primer pair QC-fw-GFPR215STOP(5′ GATCCCAACGAAAAGTGAGACCACATGGTCCTTC 3′)/ QC-rev-GFPR215STOP (5′ GAAGGACCATGTGGTCTCACTTTTCGTTGGGATC 3′).

### *B. subtilis *DB430 expression cultures

For *B. subtilis* expression cultures, 1 mL enriched LB medium in a FlowerPlate^®^ was inoculated with a single *B. subtilis* transformant and grown at 30 °C and 1100 rpm in a plate incubator (Thermomixer C, Eppendorf, Hamburg, Germany). This pre-culture was used for inoculation of 1 mL enriched LB medium in a FlowerPlate^®^ to an optical density (OD_580nm_) of 0.05, prior to cultivation at 30 °C and 1100 rpm for 24 h in a plate incubator for offline cultivation or in a BioLector microbioreactor system (m2p-labs, Baesweiler, Germany) for online cultivation and online measurements. The β-glucuronidase, encoded on pBSMul1 derivatives, was expressed under control of the strong constitutive P_*HpaII*_ promoter, whereas expression of *sfGFP* or one of the detector genes localized on pHT01 derivatives was induced by addition of 1 mM IPTG at inoculation.

### Offline GFP fluorescence measurements

For offline cultivated *B.* *subtilis* expression cultures, *sf*GFP as well as split GFP (GFP11-tag combined with the non-fluorescent detector protein) fluorescence was determined after cultivation. In vitro split GFP assay was carried out in *B. subtilis* cell lysates mixed with a GFP1-10 detector solution. GFP1-10 was produced externally by *E. coli* BL21(DE3) with pET22b-*sf*GFP1-10 in inclusion bodies as described previously [18] and solved in 100 mM Tris-HCl pH 7.4, 100 mM NaCl, 10% (v/v) glycerol, 173 mM Urea, 10 mM EDTA to obtain the detector solution. For *B. subtilis* cell lysis, 100 µl of *gus* or *gus11* expressing *B. subtilis* cultures were mixed with 25 µl PBS buffer (137 mM NaCl, 2.7 mM KCl, 8 mM Na_2_HPO_4_, 1.76 mM KH_2_PO_4_, pH 7.4) containing 10 mg/ml lysozyme. After incubation at 37 °C for at least 30 min, 20 µl cell lysates were mixed with 180 µl detector solution and were incubated at room temperature for at least 16 h as described previously [17].

For fluorescence detection of *sf*GFP in living cells or for in vivo split GFP assays (co-production of GUS11 and GFP1-10(TGA11), named “iSplit GFP assay” in this manuscript), 20 µl of the expression cultures were mixed with 180 µl PBS buffer (see above). All fluorescence measurements were carried out with a Tecan Infinite M1000 Pro microplate reader (Tecan, Männedorf, Switzerland) with the following parameters: λ_Ex_  =  485 nm (bandwidth 10 nm), λ_Em_  =  505–550 nm (5 nm steps, bandwidth 5 nm, gain 60). The emission maximum at 510 nm was used for analysis and fluorescence values were normalized to the cell density determined as optical density at 580 nm (OD_580_) for calculation of relative fluorescence units.

### Online measurements of expression cultures in a BioLector microbioreactor system

In addition to cell growth, which was analyzed via light scattering at λ  =  620 nm, the use of a BioLector microbioreactor system for cultivation of expression cultures also enabled the online monitoring of target protein production through the fluorescence signal given by the intracellular assembly of GUS11 with the co-produced detector protein. Therefore, the fluorescence was measured with the eYFP filter: λ_Ex_  =  508 nm (bandwidth 10 nm), λ_Em_  =  532 nm (bandwidth 10 nm). Measurements were carried out at a time interval of 15 min.

### Determination of β-glucuronidase activity

The enzymatic activity of GUS and GUS11 was determined with the chromogenic substrate *p*-nitrophenyl-glucuronide (*p*NPG, Sigma-Aldrich/Merck, Darmstadt, Germany) as described previously [27]. The cell lysate, generated as described above, was diluted 40-fold with PBS buffer (see above). Subsequently, 50 µl of the dilution were mixed with 50 µl substrate solution (0.5 mg/mL *p*NPG in PBS) prior to incubation at room temperature for 1 min. The reaction was stopped by addition of 100 µl 1 M Na_2_CO_3_ and the absorption at 410 nm was measured using a SpectraMax 250 plate reader (Molecular Devices, Biberach an der Riss, Germany). The volumetric activity (U/ml) was calculated using a molar absorption coefficient for *p*NP of 15,000 M^−1^ cm^−1^ for the reaction conditions used here and normalized to the cell density (OD_580nm_) as previously described [29].

### SDS-PAGE

Production of *sf*GFP and its detector derivatives was analyzed by SDS-PAGE [41]. Cells from *B. subtilis* expression cultures were harvested by centrifugation and adjusted to the requested OD_580nm_ with Tris buffer (50 mM, pH 8) before the same volume of 2  ×  SDS-sample buffer [50 mM Tris-HCl, 4% SDS, 10% (v/v) glycerol, 10% (v/v) 2-mercaptoethanol, 0.03% (w/v) bromophenol blue] was added. Proteins were separated by electrophoresis using 12% polyacrylamide gels in a Mini Protean II Dual Slap Cell (BioRad, Munich, Germany) chamber for 15 min at 100 V and for 45 min at 200 V. The separated proteins were detected using colloidal Coomassie G-250 solution [5% (w/v) ammonium sulfate, 2% (v/v) phosphoric acid, 0.02% (w/v) Coomassie Brilliant Blue G-250, 10% (v/v) ethanol], as described previously [42].

### Real-time quantitative PCR

The amount of *sfGFP*,* GFP1-10* and *GFP1-10(TGA11)* transcripts was analyzed by RT-qPCR as described previously [43]. RNA was isolated in three steps from 500 µL expression culture using the NucleoSpin^®^ RNA Kit (Macherey–Nagel, Düren, Germany), the RNase-Free DNase Set (Qiagen, Hilden, Germany) and the Ambion DNA-free™ DNA Removal Kit (Thermo Fisher Scientific, Dreieich, Germany). The cDNA was synthesized with 1 µg RNA using the Maxima First Strand cDNA Synthesis Kit (Thermo Fisher Scientific, St. Leon-Roth, Germany). RT-qPCR was performed with 50 ng cDNA using the Maxima SYBR/ROX qPCR Master Mix (Thermo Fisher Scientific, St. Leon-Roth, Germany) with the primer pairs SigA-left (5′ ATCGCCTGTCTGATCCACCA 3′)/SigA-right (5′ GGTATGTCGGACGCGGTATG 3′) for amplification of constitutively expressed major sigma factor gene *sigA* and GFP-left (5′ AAACATTCTCGGACACAAAC 3′)/GFP-right (5′ AATGGTCTGCTAGTTGAACG 3′) for amplification of *sfGFP* and the detector derivatives. Gene expression analysis was performed by using the 2^−ΔΔct^ method with an assumed PCR efficiency of 100% [44]. The expression levels of the detector genes were normalized to the level of the *sigA* gene and compared to the expression of *sfGFP*.

### Heterogeneity of reporter gene expression in *B. subtilis*

Expression heterogeneity within *B.* *subtilis* cultures was analyzed at the single-cell level by flow cytometry. Expression cultures were grown as described above and sampled when they reached the late stationary growth phase (after 24 h for *B.* *subtilis*). For this purpose, 20 µL was taken out of each culture grown in a Flowerplate^®^ and added to 600 µL PBS buffer (pH 7.4). Subsequently, the cells were harvested by centrifugation (2 min, 15,000 rpm, RT), adjusted to an optical density of 0.05 (OD_580nm_) in 100 µL PBS buffer and then transferred into a 96-well microtiter plate (Greiner Bio-One GmbH, Frickenhausen, Germany). Finally, samples were analyzed with a flow cytometer (Amnis^®^ CellStreamTM System, Luminex Corporation, Austin, USA). The individual cellular GFP fluorescence brightness was measured using a 488 nm laser (15% intensity, maximal intensity 200 mW) for excitation and a 528/46 nm bandpass filter for detection. To exclude cell debris and cell aggregates, the cells were also analyzed regarding their size (forward scatter, FSC) and granularity (side scatter, SSC). FSC was measured using an FSC laser (450 nm) with 50% of the laser power and a 456/51 nm bandpass filter for detection. For determination of SSC, a 785 nm laser with 50% of the laser power and a 773/56 nm bandpass filter was used. Based on the scatter plots, bacterial cells were gated from irrelevant counts for fluorescence analysis. Flow cytometric data were evaluated with the CellStream™ Analysis Software (Luminex Corporation, Austin, TX, USA).

### Microfluidic single-cell cultivation and live-cell imaging

Single-use Polydimethylsiloxane (PDMS) chips with microfluidic structures were used for live-cell imaging and fabricated as previously described [45, 46]. In a first step, SU-8 photolithography was performed in several layers to fabricate a structured silicon wafer, which was used as a master mold for the subsequent PDMS molding. Each PDMS microfluidic chip incorporates four separate cultivation arrays containing several hundred individual cultivation chambers each (dimensions of one cultivation chamber: 0.7 µm  ×  80 µm  ×  90 µm). The low chamber height of 0.7 µm restricts cell growth to a monolayer, enabling the accurate analysis of cell growth by image analysis with spatio-temporal resolution. All chambers are connected to 10 µm deep medium supply channels ensuring stable environmental conditions, when continuously perfused with fresh medium.

Microfluidic experiments were performed on an inverted automated microscope (Nikon Eclipse Ti, Nikon, Tokyo, Japan), equipped with a focus correction system compensating focus drift during time-lapse imaging. A benchtop incubation chamber (PECON, Erbach, Germany) ensured constant temperature conditions. The inlets to the microfluidic channels on the chip were connected to a syringe pump (neMESYS, CETONI, Korbussen, Germany) for continuous medium supply. Nikon software NIS Elements AR 4.30.02 was used for automated time-lapse imaging. The microfluidic chip was placed in an in-house fabricated chip-holder and phase contrast and fluorescence images were taken every 10 min using a 100 ×  oil immersion objective (CFI Plan Apo Lambda DM 100 × -magnification, NA 1.45). The propidium iodide fluorescence was captured through a mCherry filter (λ_ex_  =  562 nm, λ_em_  =  641 nm, DM  =  593 nm) and GFP fluorescence was captured through a GFP filter (λ_ex_  =  500 nm, λ_em_  =  542 nm, DM  =  520 nm).

Prior to chip cultivation, cells were precultured in 10 mL enriched LB medium until the OD_580nm_ reached a value of around 0.5. The cell suspension was filled into the chip with a syringe until a few cells got randomly trapped inside the cultivation chambers [47]. Subsequently, the cells were continuously perfused (flow rate 400 nL min^−1^) with fresh enriched LB medium additionally containing 1 µM propidium iodide. Propidium iodide is a fluorescent dye, which selectively enters dead cells and shows fluorescence at λ  =  617 nm after intercalation into DNA [48]. During cultivation, the chip was kept at 30 °C and images from selected chambers were taken every 10 min.

### In silico analyses

RNA secondary structure and stability predictions were performed via the Vienna RNA Websuite with RNAfold 2.4.13 [49]. The predicted MFE structures with their corresponding base pair probabilities were used for comparison of *sfGFP* and their detector derivatives’ mRNA structures. The image series generated by microfluidic experiments were processed with the image processing package Fiji [50].

## Results and discussion

### Intracellular production of GUS11 and *sf*GFP in *B. subtilis* DB430

**Fig. 1:**
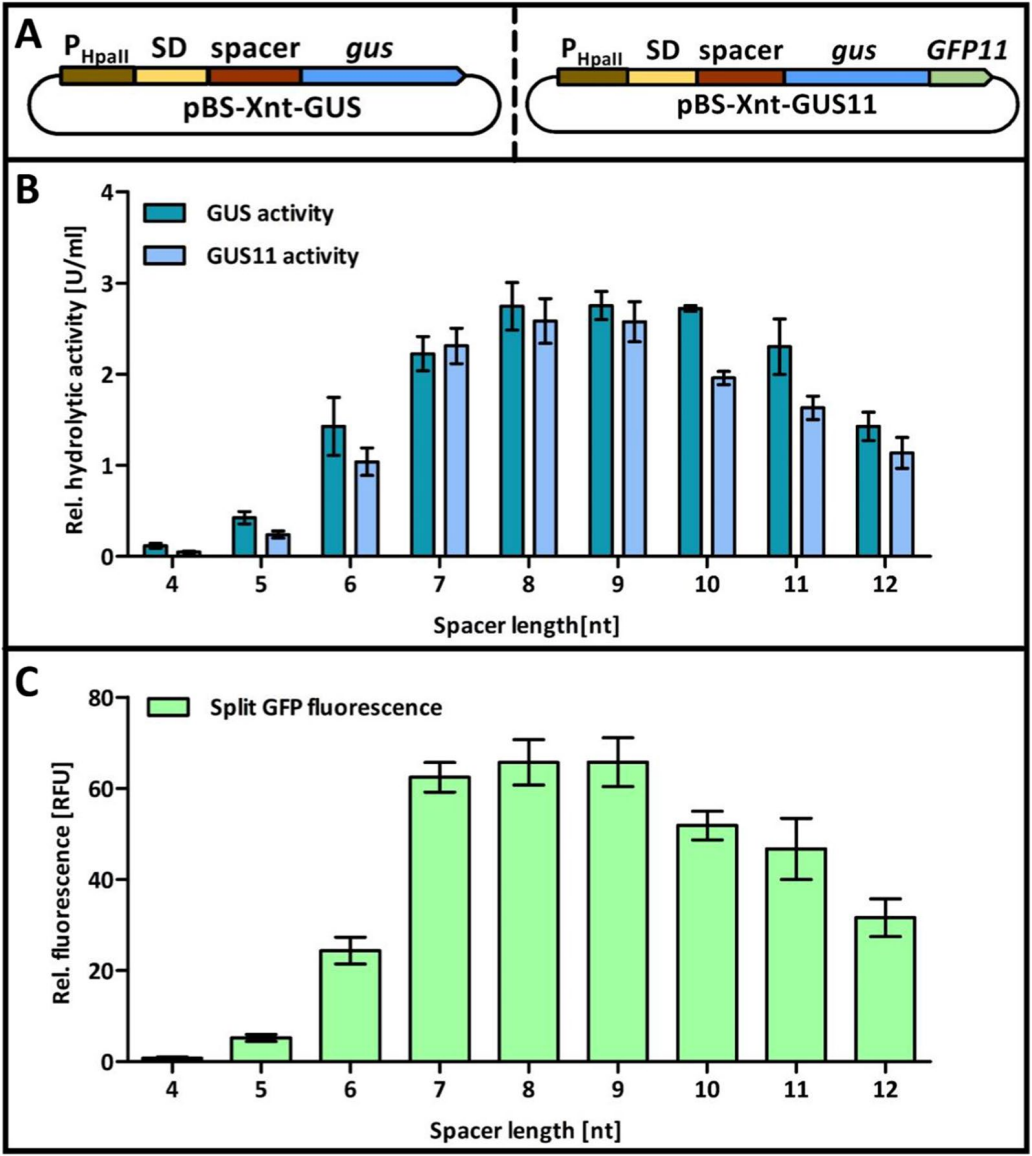
Enzymatic activity of GUS(11) and split GFP assay for the GFP11-tagged variants. β-glucuronidase was produced in different amounts by using pBS-Xnt-GUS or pBS-Xnt-GUS11 plasmid series, respectively. Plasmids of the two series harbor the strong constitutive promoter P_*HpaII*_ and differ in the length of spacer (4–12 nucleotides, indicated as Xnt in the plasmid name) located between the ribosome binding site (Shine-Dalgarno sequence, SD) and *gus* gene*.* For the pBS-Xnt-GUS11 plasmid series, a GFP11-tag encoding DNA fragment was fused to *gus* 3′ end*.* **A** Schematic depiction of GUS expression plasmids. Parts of DNA are not drawn to scale; **B** relative hydrolytic activity of GUS and GUS11; **C** fluorescence of GUS11 variants determined by split GFP assay in cell lysates of *B. subtilis* DB430. All measurements were performed in biological and technical triplicates. Error bars indicate the respective standard deviation

As a first step towards an online monitoring system for intracellular produced proteins in *B.* *subtilis*, the general applicability of the split GFP assay had to be confirmed. Therefore, the GFP11-tag was fused C-terminally to the *E.* *coli* β-glucuronidase UidA [26], here called GUS, resulting in fusion protein GUS11. The sensitivity, limits and influence of split GFP-based detection were tested by producing GFP11-tagged GUS or the native GUS at different levels using a tunable ribosome binding site with spacers consisting of 4 to 12 nucleotides [29]. GUS and GUS11 were produced in the protease deficient strain *B. subtilis* DB430 [31] carrying a plasmid of the pBS-Xnt-GUS or pBS-Xnt-GUS11 series. Both plasmid series contain the strong constitutive promoter P_*HpaII*_ for *gus* gene expression [28] and a variable spacer between the ribosome binding site and the *gus* start codon, whereby Xnt describes the spacer length (X  =  4–12 nucleotides) (Fig. 1A). The determination of the hydrolytic activity of GUS and GUS11 in cell lysates (Fig. 1B) revealed an only slight negative effect of the C-terminal GFP11 tag on GUS synthesis or activity. The results of the in vitro split GFP assay for GUS11 (Fig. 1C) largely correlated with the measured enzymatic activities. These results suggested that the easy-to-perform and inexpensive split GFP assay is also applicable as a detection method for intracellular target proteins in *B.* *subtilis* cell lysates.

As the final step towards a split GFP-based in vivo protein detection assay, target and detector proteins should be co-produced in *B. subtilis*. To analyze if the co-expression allows a comparative analysis of GUS11 accumulation inside live cells, a two-plasmid system was employed consisting of pBSMul1 [32] carrying for the gene of interest and pHT01 ([33], MoBiTec, Deutschland) encoding the detector protein. This strategy basically allows for an easy changing of the target protein without the need to change any other part of the split GFP assay system. The applicability of a two-plasmid system was initially evaluated with *B. subtilis* DB430 double transformants harboring plasmids pBS-Xnt-GUS11 for generating different amounts of intracellular GUS11 and pHT01-sfGFP to produce *sf*GFP instead of the non-fluorescent detector due to easier visualization*.* The production of GUS11 was again determined by hydrolytic activity, while the *sf*GFP amount was determined by the measurable fluorescence (see Additional file 1: Figure S1). GUS11 production remained tunable with different spacer lengths also in the presence of the second plasmid and, additionally, the *sf*GFP production was only slightly influenced by increasing GUS11 production, which confirmed the feasibility of an online monitoring system based on the expression of genes localized on two plasmids.

### Increased transcript stability enables sufficient detector production

**Fig. 2:**
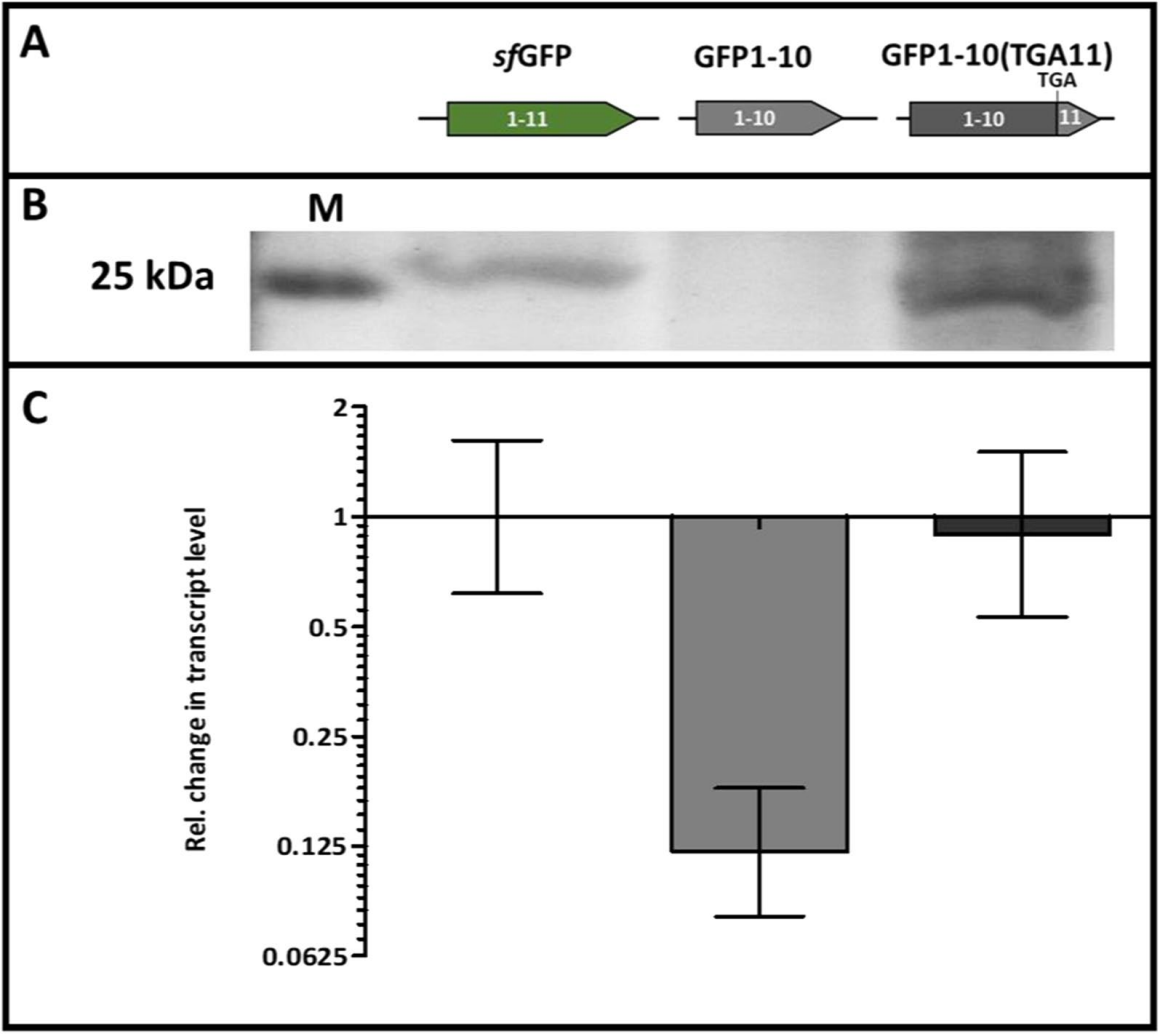
Analysis of protein and transcript accumulation for *sf*GFP and the detector variants GFP1-10 and GFP1-10(TGA11). **A** Schematic presentation of the gene variants encoding either *sf*GFP of the detector. **B** Analysis of expressed proteins by SDS-PAGE and subsequent staining with Coomassie Brilliant Blue G-250; M: PageRuler™ Prestained Protein Ladder (Thermo Fisher Scientific) was used as a marker. **C** Change of transcript amounts were determined with RT-qPCR. All samples were obtained by cultivating *B. subtilis* DB430 harboring the *sf*GFP-encoding vector pHT01-sfGFP or one of the detector plasmids pHT01-GFP1-10 or pHT01-iSplitGFP in biological and technical triplicates. Error bars indicate the respective standard deviation. Expression was induced by addition of 1 mM IPTG

Online monitoring of intracellular protein production requires sufficient co-expression of the detector protein. To this end, the *sfGFP* gene on plasmid pHT01-sfGFP was replaced by the truncated *sfGFP* gene encoding the detector protein GFP1-10 (Fig. 2A), resulting in plasmid pHT01-GFP1-10. To analyze if the expression of *sfGFP* and *GFP1-10* results in comparable expression levels, the newly constructed plasmid was introduced in *B. subtilis* DB430. After heterologous expression, intracellular proteins were separated by SDS-PAGE (Fig. 2B) and the levels of *GFP1-10* and *sfGFP* transcripts were comparatively analyzed by RT-PCR (Fig. 2C). Interestingly, in the analyzed *B. subtilis* extracts, no detector protein was observed on stained gels and the amount of transcript was eight-fold lower as compared to the full-length gene. To find a reason for these differences we further analyzed the two gene sequences. The DNA sequence of both genes is identical for the first 642 base pairs but differs in their 3′ ends as the *GFP1-10* gene is truncated by 72 base pairs. A calculation of secondary structures for both transcripts using the program RNAfold [49] revealed that the 3′-end of the *sfGFP* mRNA has a high probability of forming complex and branched structural elements. In contrast, the mRNA of the truncated *GFP1-10* gene forms a stretched single hairpin structure with a minimal free energy (MFE) reduced by approximately 1/3 (see Additional file 1: Figure S2). This observation suggests that the more simple structure of the *GFP1-10* transcript may promote degradation by RNase III, whereas the more complex secondary structural elements observed for the *sfGFP* transcript may inhibit its degradation, e.g., by masking RNase recognition sites (reviewed in [51]).

Thus, a new detector construct was established by introducing a stop codon into the *sfGFP* gene at a position behind the DNA sequence encoding the tenth β-sheet instead of fully deleting this part of the gene (Fig. 2A): this construct thus encodes the same GFP1-10 protein but minimally affects the mRNA´s secondary structure. The resulting *GFP1-10(TGA11)* transcript has a high probability of forming branched structural elements, which results in a MFE comparable to the *sfGFP* transcript (see Additional file 1: Figure S2). Remarkably, the amount of transcript of this *GFP1-10(TGA11)* gene encoded on plasmid pHT01-iSplitGFP was comparable to the amount of *sfGFP* transcript (Fig. 2C) and, additionally, a corresponding protein band was detected by SDS-PAGE and staining (Fig. 2B).

### Plasmid pHT01-iSplitGFP enables in vivo detection of intracellular target proteins

**Fig. 3:**
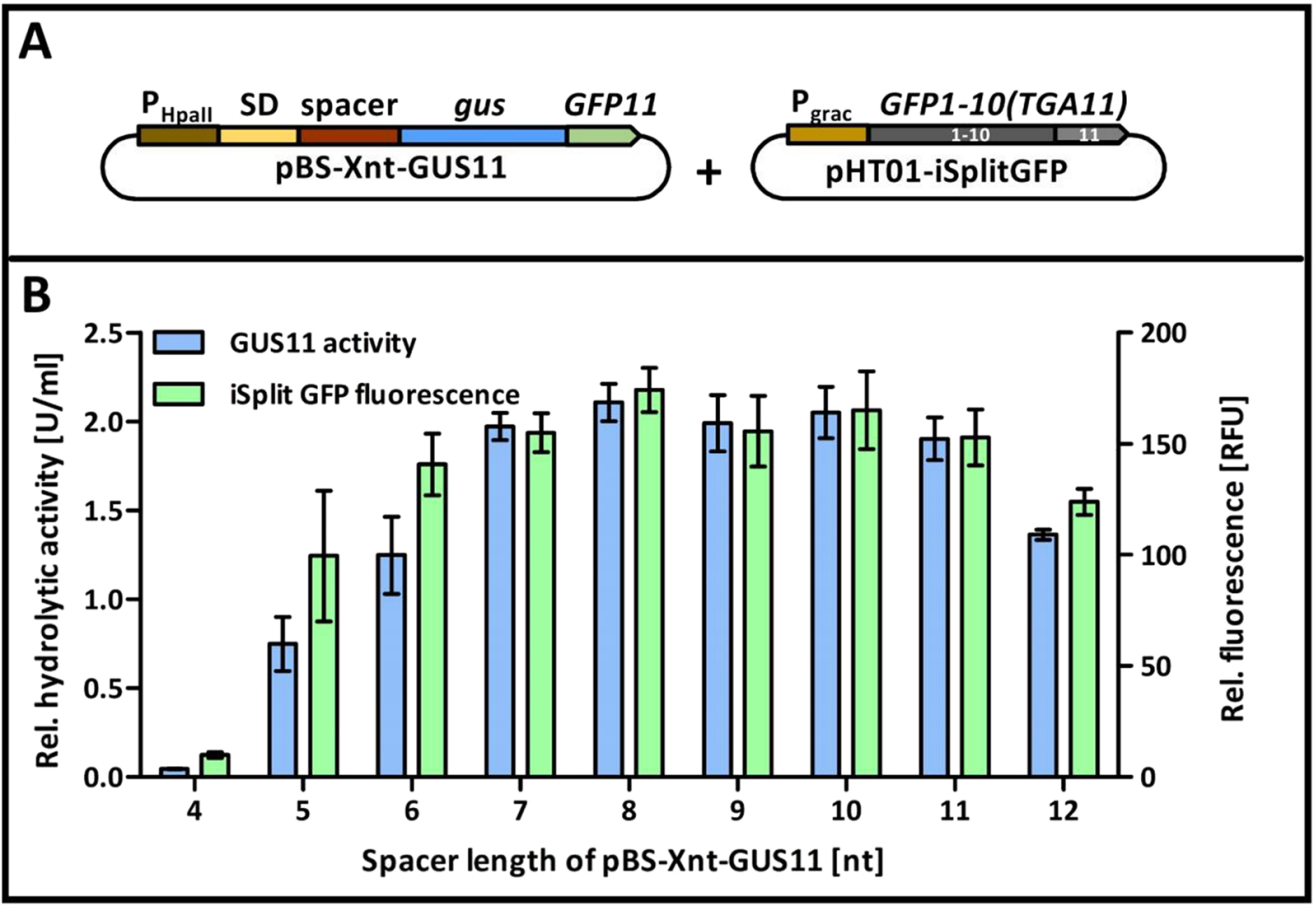
Gradual production of GUS11 determined as enzymatic activity and iSplit GFP assays. *B.* *subtilis* DB430 was transformed with pBS-Xnt-GUS11 plasmids coding for GUS11 and harboring the strong constitutive promoter P_*HpaII*_ and ribosome binding site spacers of variable length (4 to 12 nucleotides; indicated by Xnt in plasmid name) and with the GFP1-10(TGA11) detector encoded on plasmid pHT01-iSplitGFP harboring the IPTG inducible promoter P_*grac*_. **A** Schematic presentation of plasmid construct. Parts of DNA are not drawn to scale; **B** relative enzymatic activity and iSplit GFP fluorescence of GUS11 were measured in biological and technical triplicates. Error bars indicate the respective standard deviation. The expression of detector protein was induced by addition of 1 mM IPTG

As a next step towards a split GFP-based in vivo protein detection assay, target and detector proteins were co-produced in *B.* *subtilis* from the newly constructed plasmid pHT01-iSplitGFP and a pBS-Xnt-GUS11 series plasmid (Fig. 3A). After cultivation, the production of GUS11 was quantified either using cell lysates for determination of enzymatic activity with *p*NPG as substrate or by measuring split GFP fluorescence in living cells resulting from GUS11 interaction with the co-produced GFP1-10(TGA11) detector protein (Fig. 3B). The data obtained by both assays showed a good correlation as observed previously with the in vitro assay (Fig. 1), indicating that the iSplit GFP assay can be used to monitor the intracellular GUS11 formation in vivo*.*

### Online monitoring of GUS11 formation during cell cultivation using iSplit GFP assay

**Fig. 4:**
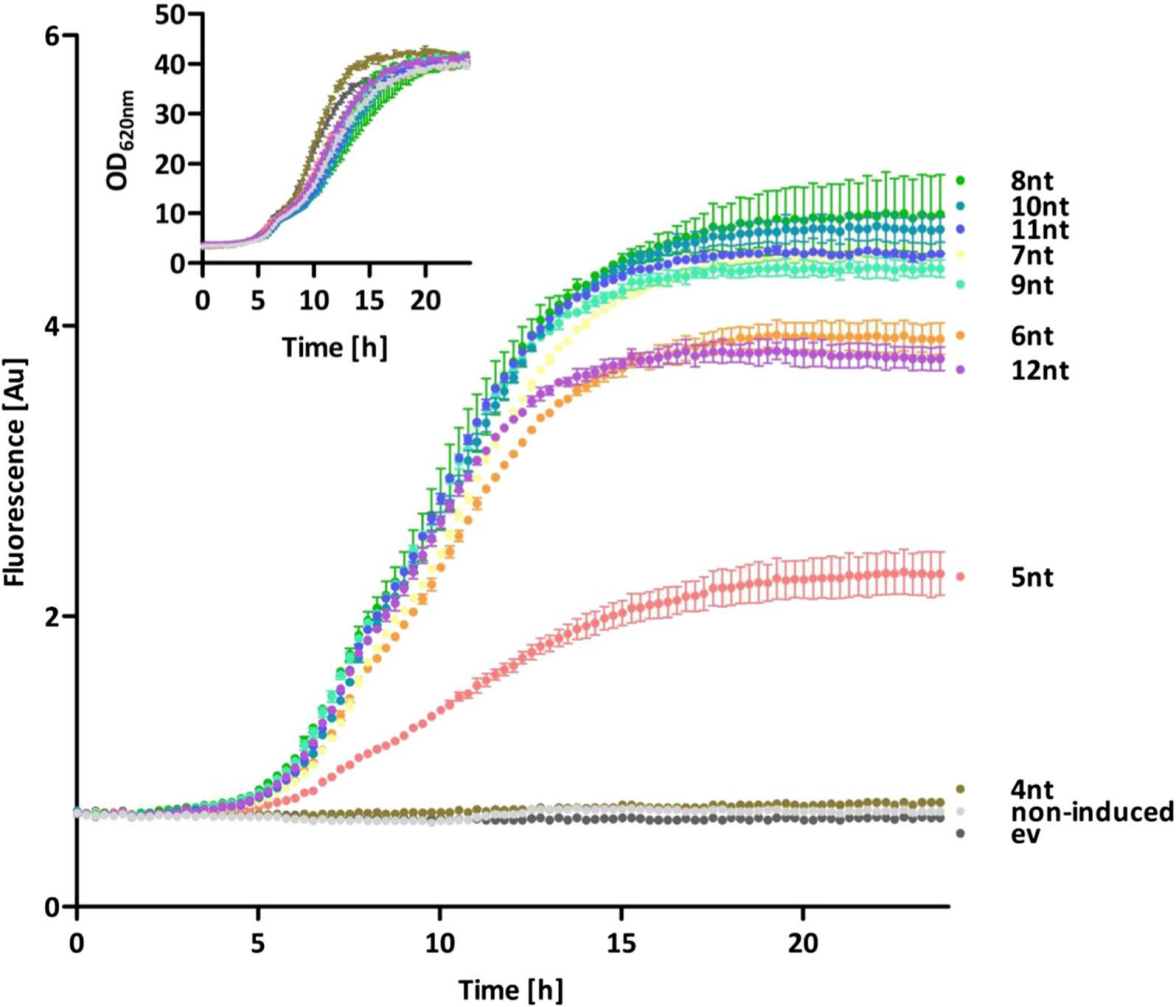
Growth of *B.* *subtilis* and online measurement of differential GUS11 production using iSplit GFP assay. For measuring growth and GUS11 production online, a BioLector microbioreactor system was used. iSplit GFP fluorescence and cell density (determined by light scattering at λ  =  620 nm) were measured of *B.* *subtilis* DB430 harboring pHT01-iSplitGFP for detector production and a pBS-Xnt-GUS11 plasmid with different spacers (4–12 nt; indicated by Xnt in plasmid series name) for GUS11 production or the associated empty vector (ev). For detector expression, cultures were supplemented with 1 mM IPTG at inoculation. As negative controls both, the empty vector pBSMul1 (ev) and a pBS-8nt-GUS11 sample, whose detector expression was not induced (non-induced), were included. Cultivation was carried out in biological and technical triplicates with error bars indicating the standard deviation

For online monitoring of cell growth and target protein formation with the iSplit GFP assay, *B. subtilis* was cultivated in a BioLector microbioreactor system. *B. subtilis* DB430 carrying one of the pBS-Xnt-GUS11 plasmids and, additionally, pHT01-iSplitGFP was cultivated with 1 mM IPTG at 30 °C for 24 h, while cell density and fluorescence were continuously measured (Fig. 4). All GUS11 and detector producing cultures showed similar growth behavior (Fig. 4, inset), therefore, the fluorescence values determined for different strains with the iSplit GFP assay could directly be compared with one another. For all strains, the GUS11 production reached a plateau when the cultures entered the stationary growth phase (after approximately 15 h), presumably because the constitutive promoter P_*HpaII*_ used here is active only during the exponential growth phase [28]. As expected, the GUS11 expressing strains differed in their fluorescence intensities according to the varying spacer lengths and thus corroborating previous results (Figs. 1, 3). The different GUS11 formation observed in the iSplit GFP assay was additionally confirmed by an activity measurement of the cell lysates (see Additional file 1: Figure S3). The iSplit GFP assay thus proved suitable as an online monitoring system for the detection of intracellular target protein production during *B. subtilis* cultivation*.*

### iSplit GFP assay indicates recombinant protein production at the single-cell level

**Fig. 5:**
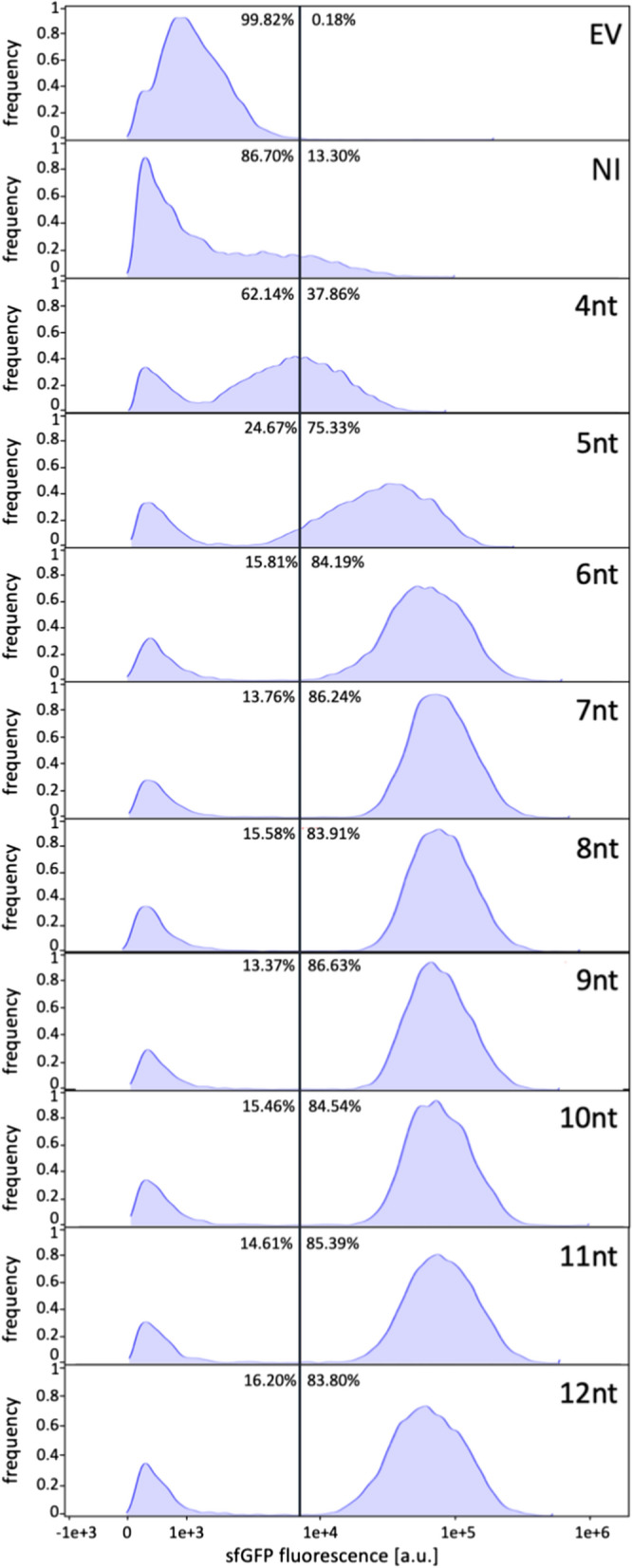
Fluorescence distribution of single *B. subtilis* cells producing varying amounts of GUS11 analyzed by flow cytometry. Cultures of *B.* *subtilis* DB430 harboring plasmids pBS-Xnt-GUS11 and pHT01-iSplitGFP for expression of *gus11* with varying spacers from 4 to 12 nucleotides (as indicated by Xnt) and the detector protein were grown at 30 °C. For the induction of detector gene expression cultures were supplemented with 1 mM IPTG prior to inoculation. As negative controls, both an empty vector control (EV) and a pBS-8nt-GUS-11 variant without induction of detector expression (NI) were included. Culture samples were collected at the late stationary growth phase (after 24 h) and analyzed by flow cytometry. The cells were gated based on their respective FSC and SSC signals to exclude cell debris and accumulation of cells (see Additional file 1: Figure S4). The iSplit GFP fluorescence intensity of each cell was measured and plotted against the frequency of the signal intensities. The percentages of fluorescent to non-fluorescent cells separated by a line are shown in each graph. All graphs are representative examples of triplicate measurements

Since *B. subtilis* can adopt different cellular states, population heterogeneity is an issue also for the production of recombinant proteins [52]. Such heterogeneity can be studied by single-cell analyses like flow cytometry or microfluidics. As these analyses are often used in combination with fluorescence reporters, the iSplit GFP assay should have potential as a tool for monitoring population heterogeneity with respect to target protein production. To investigate this, expression cultures of the strain *B. subtilis* DB430 carrying the vector series pBS-Xnt-GUS11 and pHT01-iSplitGFP induced with 1 mM IPTG were grown for 24 h until late logarithmic growth phase in a BioLector microbioreactor and fluorescence intensity and fluorescence distribution of 10,000 cells were analyzed by flow cytometry (Fig. 5). The analyzed cells were gated based on their light scattering properties to exclude cell debris and cell aggregates (see Additional file 1: Figure S4 and method section “Heterogeneity of reporter gene expression in *B. subtilis*”).

**Fig. 6:**
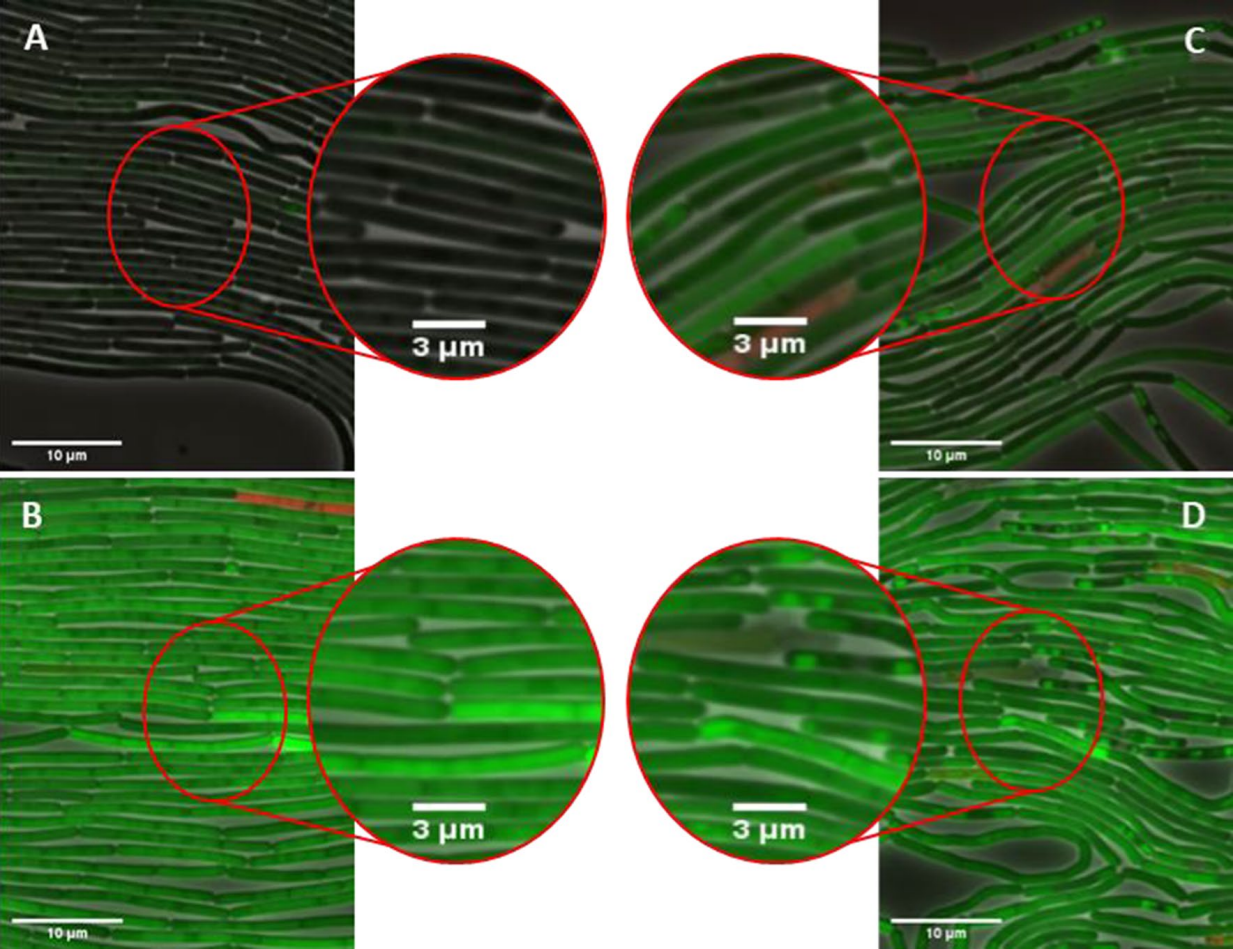
Cytoplasmic distribution of GUS11 in *B. subtilis* cells detected in vivo by iSplit GFP assay. Microfluidic chambers were inoculated with *B. subtilis* DB430 harboring pHT01 and pBSMul1 empty vector **A** or pHT01-sfGFP and pBSMul1 **B** as controls. *Bacilli* harboring pHT01-iSplitGFP and one of the *gus11* expression vectors, pBS-4nt-GUS11 **C** or pBS-8nt-GUS11 **D**, respectively. Overall growth was analyzed by phase contrast microscopy. GUS11 formation and *sf*GFP production were detected with a GFP filter whereas dead cells were detected by using propidium iodide and an mCherry filter. All three channels were merged. For better visibility, the image was zoomed in at representative points. Cells were cultivated in optimized LB medium with 1 mM IPTG and 1 µM propidium iodide at 30 °C for approximately 18 h

The results obtained by flow cytometry resemble those obtained by monitoring bulk cultures (Figs. 3, 4): with increasing spacer length of 4–9 nucleotides, the fluorescence intensities of the individual populations also increased until a plateau was reached at a spacer length of 7 nucleotides, which remained almost constant also for longer spacers. From a spacer length of 6 nucleotides onwards, 75–85% of all cells constantly exhibit similar fluorescence levels indicating that the GUS11 does not trigger production heterogeneity. For spacers with only 4 or 5 nucleotides, which result in low translation initiation rates, a relatively large heterogeneity was observed as represented by a broad distribution of fluorescence intensity. This might be explained by the fact that ribosome occupancy of mRNA is less likely at low translation initiation rates thereby promoting mRNA degradation and thus probably production heterogeneity (reviewed in [53]). This assumption is supported by the fact that differences in mRNA quantity were observed for different spacer lengths [29]. In addition to heterogeneity, decreased GUS11 production was detected by the generally lower fluorescence intensities for these variants compared to the longer spacers. This phenomenon was previously explained by rigid steric effects of mRNA affecting binding to the ribosome [29, 54]. However, in the case of low translation initiation rates, production heterogeneity could possibly play a more important role. The strain harboring the plasmid with the 8 nucleotide spacer showed the lowest variance within the fluorescence intensity. We therefore selected this strain and the strain harboring pBS-4nt-GUS11 as the most heterogeneous one for further characterization by microfluidic single-cell analysis in combination with live-cell microcopy. Time-lapse imaging can be used to visualize the intracellular distribution of a target protein and the effects of its production on the cell morphology. We cultivated both variants together with suitable controls in a microfluidic system and analyzed cell growth, morphology, GUS11 production by split GFP fluorescence and also cell lysis by a propidium iodide assay [48] (Fig. 6).

*B. subtilis* cells showed a filamentous appearance during the exponential growth phase (see Additional files 2, 3, 4, 5: V1–4) as previously described [55]. Additionally, it was possible to discriminate live and dead cells by using the PI assay. As expected, the negative control, a double transformant harboring both empty vectors, showed neither GFP fluorescence nor significant cell lysis as indicated by the absence of PI fluorescence (Fig. 6A; see Additional file 2: V1). The GFP fluorescence of the positive control showed a relatively homogenous distribution both within the population and within the cytoplasm of individual cells, with some population heterogeneity observable. The cell lysis rate was comparable to the negative control (Fig. 6B; see Additional file 3: V2). As expected, the overall fluorescence intensity and homogeneity were higher in the variant with an 8nt spacer than in the 4nt variant thereby corroborating the flow cytometry data (Fig. 5). Notably, in contrast to cells producing *sf*GFP, the fluorescence obtained by the iSplit GFP assay is disparately distributed as indicated by fluorescence spots within the cytoplasm (Fig. 6C, D; see Additional file 4: V3, V4). In *E.* *coli*, protein aggregates were detected by the in vivo split GFP assay [19, 20], suggesting that the fluorescence spots observed here may consist of aggregates formed by GUS11. Such aggregates partly form inclusion bodies, which are known to be located at the cell poles in *E.* *coli* [56, 57]. Upon expression of the 8nt variant an only slightly increased number of dead cells was detected with PI indicating that the iSplit GFP only marginally affected the cell viability.

Our data demonstrate that the iSplit GFP assay can be used for online monitoring of recombinant protein expression in bulk cultures, but also for analyzing population heterogeneity, intracellular protein distribution, and target protein aggregation without affecting cell viability.

## Conclusions

The Gram-positive bacterium *B. subtilis* is one of the work horses in industrial biotechnology, mainly due to its potent secretion machinery, which is capable of secreting up to 20 g/L protein [1]. Additional advantages include the absence of endotoxic lipopolysaccharides, the GRAS classification by the FDA [2], and the lack of a codon bias [4]. Until now, the production of intracellular recombinant proteins by *B.* *subtilis* has not been thoroughly explored, presumably because they are difficult to detect and quantify.

In this study, we established the iSplit GFP assay for the detection of intracellular target proteins in *B. subtilis*. Based on results recently reported for *Corynebacterium glutamicum* and *E.* *coli*, fluorescent biosensors can be used for screening of large libraries by fluorescence-activated cell sorting (FACS) [58, 59]. It is therefore reasonable to assume that the iSplit GFP assay can be adapted for high throughput detection of intracellular protein production by *B.* *subtilis*. Furthermore, the iSplit GFP assay allows for microfluidic single-cell analysis, which can serve to detect population heterogeneity and to localize intracellular proteins. In summary, the iSplit GFP system described here is a novel tool, which significantly facilitates monitoring of intracellular protein production by *B.* *subtilis.* It could be used in future studies that intentionally aim for intracellular production but potentially also for secretory protein production strategies to monitor the proportion of produced protein that remain in the cytosol.

## Supplementary Information

**Additional file 1: Figure S1.** Enzymatic activity of GUS11 and *sf*GFP fluorescence in a two-plasmid system.* B. subtilis *DB430 was transformed with pBS-Xnt-GUS11 plasmids harboring the *gus11 *gene with different upstream located spacer sequences (4–12 nucleotides, indicated by Xnt in plasmid name) and the strong constitutive promoter P_*HpaII*_ and with *sfGFP* encoded on plasmid pHT01-sfGFP harboring the with IPTG inducible promoter P_*grac*_. (**A**) Schematic presentation of plasmid constructs; DNA fragments are not drawn to scale (**B**) relative hydrolytic activity of GUS11 and *sf*GFP fluorescence in biological and technical triplicates. The error bars represent the corresponding standard deviation. The expression of the *sfGFP* gene was induced by addition of 1 mM IPTG. **Figure S2.** Calculated minimum free energy (MFE) mRNA structures and energies of sfGFP and the detector variants GFP1-10 and GFP1-10(TGA11). The MFE structures and energies were calculated with the Vienna Websuite based on RNAfold [49]. In addition to the energies of the entire structures, the energies of the different 3′-ends were calculated separately (circled area). The structure propability is displayed with a color gradient from violet to red equivalent to a probability of 0–1. **Figure S3.** Differential production of GUS11 determined as enzymatic activity after growth of cultures in a BioLector microbioreactor system. *B.* *subtilis* DB430 was transformed with one plasmid of the pBS-Xnt-GUS11 plasmid series which is coding for GUS11 and harboring the strong constitutive promoter P_*HpaII*_ and ribosome binding site spacers of different length (4–12 nucleotides, indicated by Xnt in plasmid name) and with the GFP1-10(TGA11) expression plasmid pHT01-iSplitGFP also harboring the with IPTG inducible promoter P_*grac*_. Cultivation was conducted in a BioLector microbioreactor for 24 h (growth and fluorescence online measurements from these cultures are shown in Fig. 4). (**A**) Schematic presentation of plasmid constructs; (**B**) Relative GUS11 activities detected in B. subtilis cultivated for 24 h in a BioLector microbioreactor system. The here shown data were gained with the same cultures whose iSplit GFP fluorescence is shown in Fig. 4. Data represent mean values of biological and technical triplicates and error bars indicate the respective standard deviations. The expression of GFP1-10(TGA11) was induced by addition of 1 mM IPTG. As iSplit GFP negative controls, both, the empty vector pBSMul1 (ev) and a pBS-8nt-GUS11 sample without induction of detector expression (NI) were included. **Figure S4.** Light scattering properties of *B. subtilis* DB430 double transformants determined by flow cytometry. Scatter of side versus forward scatter of *B.* *subtilis* DB430 cells harboring plasmids pBS-Xnt-GUS11 and pHT01-iSplitGFP for expression of *gus11* with varying spacers from 4 to 12 nucleotides (as indicated by Xnt) and the detector protein, to exclude cell debris and cell aggregates. The cells of interest, which were gated are colored in blue. The analyzed cells were grown at 30 °C and supplemented with 1 mM IPTG prior to cultivation. As negative control, both an empty vector control (EV) and the non-induced pBS-8nt-GUS11 variant (NI) were included. All graphs are representative examples of triplicate measurements.

**Additional file 2: V1.** Microfluidic cultivation of *B. subtilis *DB430 cells harboring both empty vectors. Time-lapse video of a microfluidic cultivation chamber with growing *B. subtilis *DB430 cells harboring pBSMul1 and pHT01 empty vectors. Cells were supplied with enriched LB medium, 1 mM IPTG and 1 µM propidium iodide. During cultivation, the chip was kept at 30 °C and images were taken in an interval of 10 min. Overall growth was analyzed by phase contrast (gray). *sf*GFP fluorescence was detected with a GFP filter (green), and cell death was detected using propidium iodide and an mCherry filter (red). All three channels were merged for easier visualization.

**Additional file 3: V2.** Microfluidic cultivation of *sf*GFP producing *B. subtilis *DB430 cells. Time-lapse video of one microfluidic cultivation chamber with growing *B. subtilis *DB430 cells harboring pBSMul1 empty vector and *sf*GFP encoding plasmid pHT01-sfGFP. Cells were supplied with enriched LB medium, 1 mM IPTG and 1 µM propidium iodide. Cultivation was performed at 30 °C and images were taken in an interval of 10 min. Overall growth was analyzed by phase contrast (gray). *sf*GFP fluorescence was detected with a GFP filter (green), and cell death was detected by propidium iodide and an mCherry filter (red). All three channels were merged for easier visualization.

**Additional file 4: V3.** Microfluidic cultivation of low-level GUS11 producing *B. subtilis *DB430 cells visualized by iSplit GFP assay. Time-lapse video of one microfluidic cultivation chamber with growing *B. subtilis *DB430 cells harboring pBS-4nt-GUS11 coding for GUS11 with a 4 nucleotide spacer and the GFP1-10(TGA11) encoding plasmid pHT01-iSplitGFP. Cells were supplied with enriched LB medium, 1 mM IPTG and 1 µM propidium iodide. Images were taken in an interval of 10 min. Cultivation was performed at 30 °C. Overall growth was analyzed using phase contrast (gray). GUS11 formation was detected with a GFP filter (green), and cell death was detected using propidium iodide and an mCherry filter (red). All three channels were merged for easier visualization.

**Additional file 5: V4.** Microfluidic cultivation of high-level GUS11 producing *B. subtilis *DB430 cells visualized using iSplit GFP assay. Time-lapse video of one microfluidic cultivation chamber with growing *B. subtilis *DB430 cells harboring pBS-8nt-GUS11 coding for GUS11 with an 8 nucleotide spacer and the GFP1-10(TGA11) encoding plasmid pHT01-iSplitGFP. Cell were supplied with enriched LB medium, 1 mM IPTG and 1 µM propidium iodide. Through cultivation, the chip was kept at 30 °C and images were taken in an interval of 10 min. Overall growth was analyzed using phase contrast (gray). GUS11 formation was detected with a GFP filter (green), and cell death was detected by propidium iodide and an mCherry filter (red). All three channels were merged for easier visualization.

## Data Availability

All data generated or analyzed during this study are included in this article and its additional files.
