# Supplementary material for: The iSplit GFP assay detects intracellular recombinant proteins in Bacillus subtilis
Source: Microb Cell Fact. 2021 Sep 6;20:174. doi: 10.1186/s12934-021-01663-7 (PMC8419962; doi:10.1186/s12934-021-01663-7)
Supplement: Supplementary file 1 — Additional file 1: Figure S1. Enzymatic activity of GUS11 and sfGFP fluorescence in a two-plasmid system. B. subtilis DB430 was transformed with pBS-Xnt-GUS11 plasmids harboring the gus11 gene with different upstream located spacer sequences (4–12 nucleotides, indicated by Xnt in plasmid name) and the strong constitutive promoter PHpaII and with sfGFP encoded on plasmid pHT01-sfGFP harboring the with IPTG inducible promoter Pgrac. (A) Schematic presentation of plasmid constructs; DNA fragments are not drawn to scale (B) relative hydrolytic activity of GUS11 and sfGFP fluorescence in biological and technical triplicates. The error bars represent the corresponding standard deviation. The expression of the sfGFP gene was induced by addition of 1 mM IPTG. Figure S2. Calculated minimum free energy (MFE) mRNA structures and energies of sfGFP and the detector variants GFP1-10 and GFP1-10(TGA11). The MFE structures and energies were calculated with the Vienna Websuite based on RNAfold [49]. In addition to the energies of the entire structures, the energies of the different 3′-ends were calculated separately (circled area). The structure propability is displayed with a color gradient from violet to red equivalent to a probability of 0–1. Figure S3. Differential production of GUS11 determined as enzymatic activity after growth of cultures in a BioLector microbioreactor system. B. subtilis DB430 was transformed with one plasmid of the pBS-Xnt-GUS11 plasmid series which is coding for GUS11 and harboring the strong constitutive promoter PHpaII and ribosome binding site spacers of different length (4–12 nucleotides, indicated by Xnt in plasmid name) and with the GFP1-10(TGA11) expression plasmid pHT01-iSplitGFP also harboring the with IPTG inducible promoter Pgrac. Cultivation was conducted in a BioLector microbioreactor for 24 h (growth and fluorescence online measurements from these cultures are shown in Fig. 4). (A) Schematic presentation of plasmid constructs; (B) Relative GU [file 12934_2021_1663_MOESM1_ESM.pdf]

Additional file 1

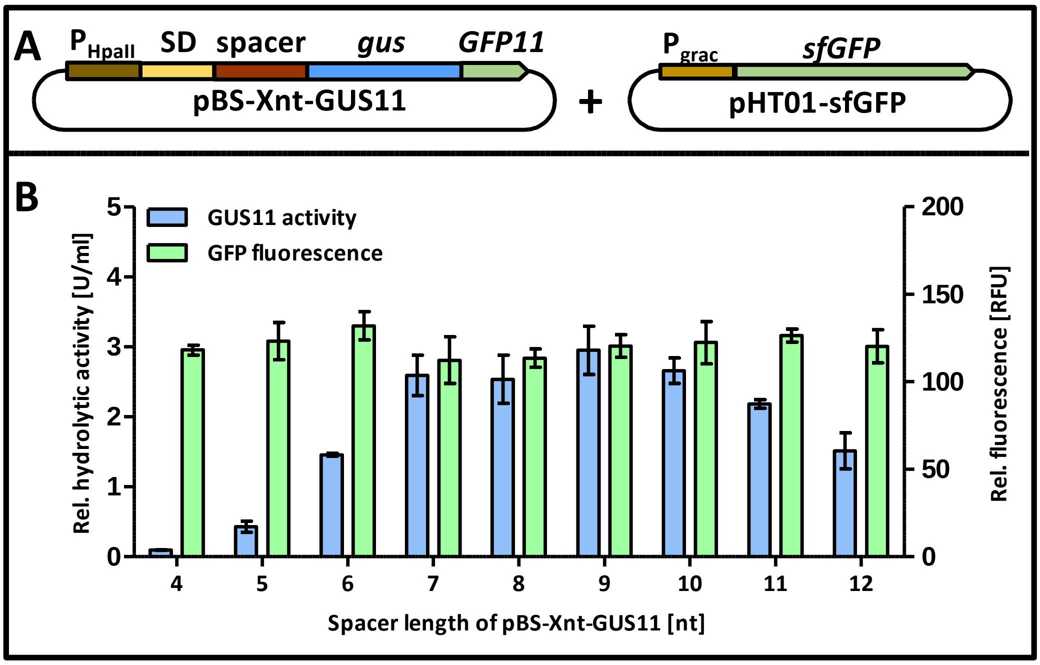

Fig. S1 Enzymatic activity of GUS11 and *sf*GFP fluorescence in a two-plasmid system. *B. subtilis* DB430 was transformed with pBS-Xnt-GUS11 plasmids harboring the *gus11* gene with different upstream located spacer sequences (4 to 12 nucleotides, indicated by Xnt in plasmid name) and the strong constitutive promoter $_{PHpaII}$ and with *sfGFP* encoded on plasmid pHT01-sfGFP harboring the with IPTG inducible promoter P_{grac}. (**A**) Schematic presentation of plasmid constructs; DNA fragments are not drawn to scale (**B**) relative hydrolytic activity of GUS11 and *sf*GFP fluorescence in biological and technical triplicates. The error bars represent the corresponding standard deviation. The expression of the *sfGFP* gene was induced by addition of 1 mM IPTG.

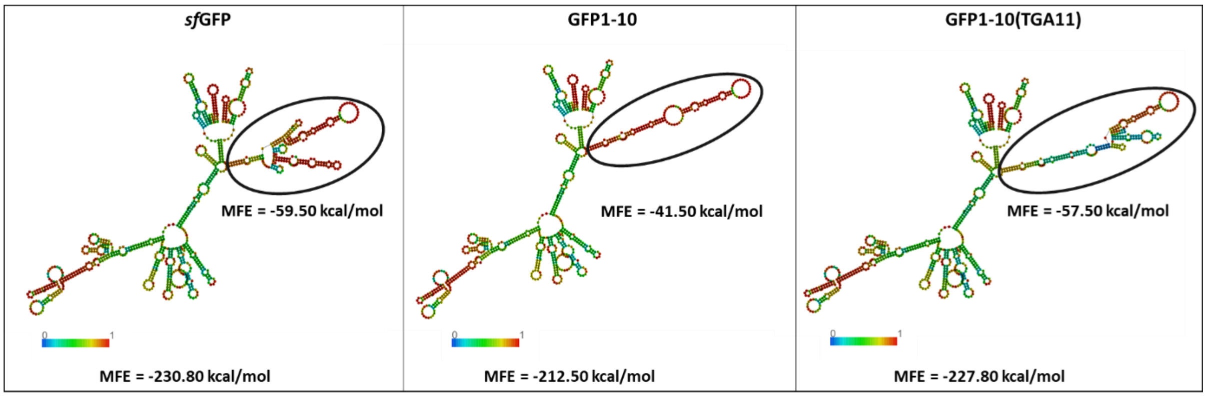

Fig. S2 Calculated minimum free energy (MFE) mRNA structures and energies of *sfGFP* and the detector variants *GFP1-10* and *GFP1-10(TGA11)*. The MFE structures and energies were calculated with the Vienna Websuite based on RNAfold [49]. In addition to the energies of the entire structures, the energies of the different 3'-ends were calculated separately (circled area). The structure propability is displayed with a color gradient from violet to red equivalent to a probability of 0 to 1.

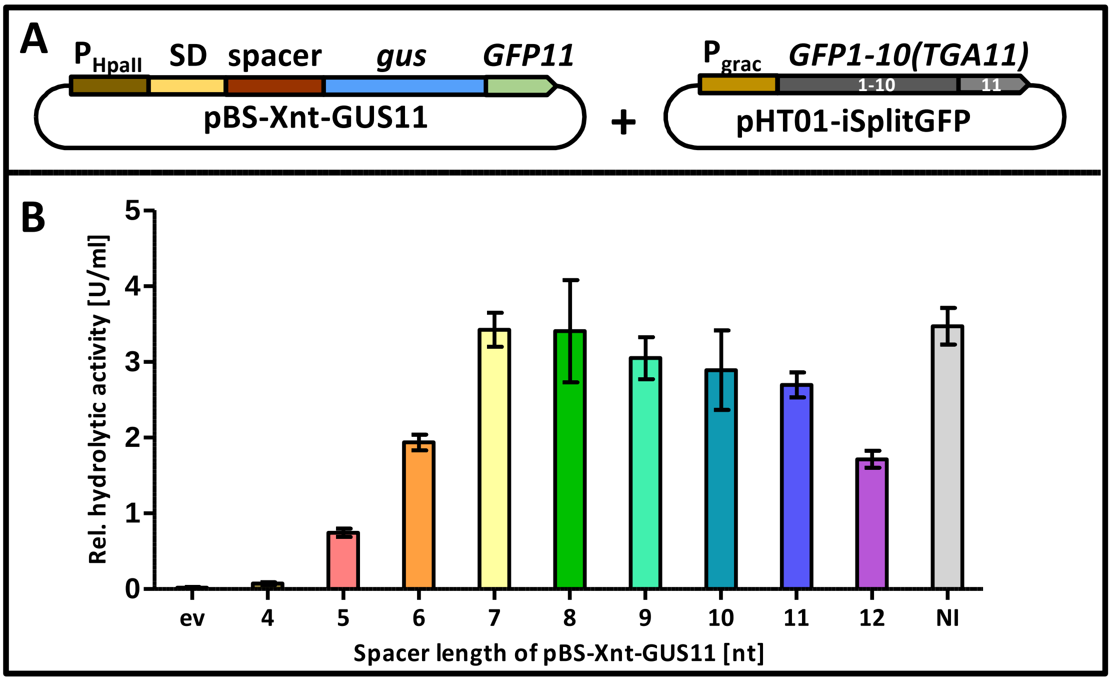

Fig. S3 Differential production of GUS11 determined as enzymatic activity after growth of cultures in a BioLector microbioreactor system. *B. subtilis* DB430 was transformed with one plasmid of the pBS-Xnt-GUS11 plasmid series which is coding for GUS11 and harboring the strong constitutive promoter $_{PHpaII}$ and ribosome binding site spacers of different length (4 to 12 nucleotides, indicated by Xnt in plasmid name) and with the *GFP1-10(TGA11)* expression plasmid pHT01-iSplitGFP also harboring the with IPTG inducible promoter P_{grac}. Cultivation was conducted in a BioLector microbioreactor for 24 h (growth and fluorescence online measurements from these cultures are shown in Fig. 4). (**A**) Schematic presentation of plasmid constructs; (**B**) Relative GUS11 activities detected in *B. subtilis* cultivated for 24 h in a BioLector microbioreactor system. The here shown data were gained with the same cultures whose iSplit GFP fluorescence is shown in Fig. 4. Data represent mean values of biological and technical triplicates and error bars indicate the respective standard deviations. The expression of *GFP1-10(TGA11)* was induced by addition of 1 mM IPTG. As iSplit GFP negative controls, both, the empty vector pBSMul1 (ev) and a pBS-8nt-GUS11 sample without induction of detector expression (NI) were included.

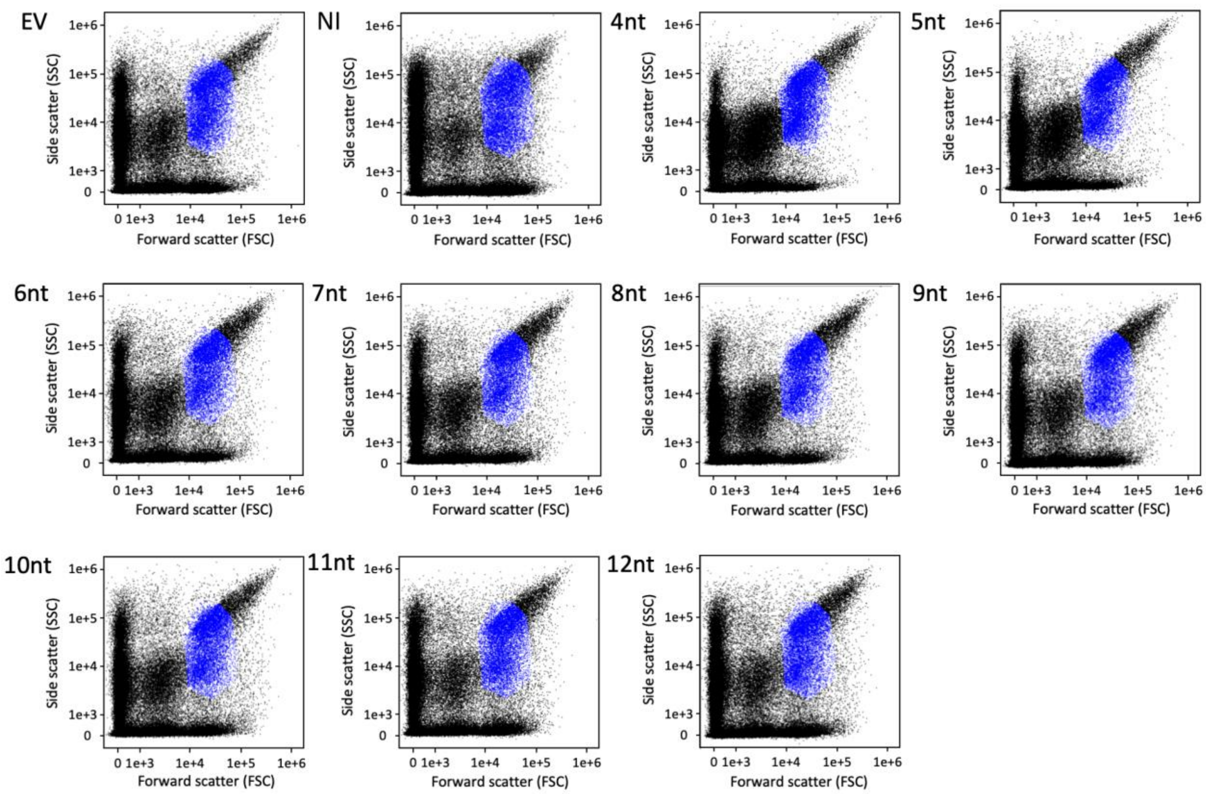

Fig. S4: Light scattering properties of *B. subtilis* DB430 double transformants determined by flow cytometry. Scatter of side versus forward scatter of *B. subtilis* DB430 cells harboring plasmids pBS-Xnt-GUS11 and pHT01-iSplitGFP for expression of *gus11* with varying spacers from 4 to 12 nucleotides (as indicated by Xnt) and the detector protein, to exclude cell debris and cell aggregates. The cells fo interest, which were gated are colored in blue. The analyzed cells were grown at 30 °C and supplemented with 1 mM IPTG prior to cultivation. As negative control, both an empty vector control (EV) and the non-induced pBS-8nt-GUS11 variant (NI) were included. All graphs are representative examples of triplicate measurements.

References

1. Gruber AR, Lorenz R, Bernhart SH, Neuböck R, Hofacker IL: **The Vienna RNA Websuite.** *Nucleic Acids Research* 2008, **36:**W70-W74.
